# A flexible heat pump cycle for heat recovery

**DOI:** 10.1038/s44172-022-00018-3

**Published:** 2022-08-01

**Authors:** Zhibin Yu, Andrew McKeown, Zahra Hajabdollahi Ouderji, Miryam Essadik

**Affiliations:** James Watt School of Engineering, University of Glasgow, Glasgow, G12 8QQ UK

**Keywords:** Energy modelling, Mechanical engineering

## Abstract

Heat pumps will play a key role in transitioning domestic heating to fossil-free sources. However, improvement in energy efficiency and cost reduction are still needed. Current vapour-compression heat pumps are built upon the Evans-Perkins cycle which was originally designed for refrigeration applications. Once hot liquid refrigerant has transferred energy to the central heating system, it leaves the condenser with sensible heat which can be utilized. Here we report a modified and flexible Evans-Perkins heat pump cycle integrating heat recovery and storage which is then used as an ancillary heat source for the heat pump’s operation. It operates in a quasi-two-stage mode to theoretically save up to 20% in compressor power consumption compared with single-stage cycles. We build a prototype with off-the-shelf parts and demonstrate a practical 3.7% power saving at a heat production temperature of 35 °C. Power saving will further increase with heat supply temperature. We also qualitatively show that hot refrigerant exiting the condenser can be directly used for defrosting the evaporator, providing additional energy saving.

## Abstract (web summary)

Yu and colleagues report a modified and flexible Evans-Perkins heat pump cycle integrating heat recovery and storage and demonstrate its application in a prototype heat pump. The recovered heat can be used as an ancillary heat source for the heat pump’s operation and to defrost the heat pump evaporator as necessary.

## Introduction

Total heat energy consumed by domestic and industrial applications accounts for almost half of global final energy consumption in 2021, contributing more than 40% (13.1 Gt) of global energy-related CO_2_ emissions in 2020^1^. To meet the net-zero emissions target by 2050, 600 million heat pumps should be installed annually by 2030^2^. As of 2020, only 180 million are used globally, accounting for 7% of building demand^2^. Among heat pump products, air source heat pumps (ASHP) are the cheapest, but still remain 3–4 times more expensive than a gas boiler both in the UK and Germany^3^. Improvement in energy performance and cost reduction are critical to facilitate the uptake of heat pumps to replace fossil fuel-based products^4–6^.

The heating capacity and Coefficient of Performance (COP) of ASHPs decrease as the outdoor temperature drops. Therefore, most ASHPs require an auxiliary heating system^7^. The research community has made continuous efforts to develop new methods to improve heat pumps’ performance, particularly for applications with high temperature lift.

One way to address this challenge is to use multi-stage heat pumps. They compress the refrigerant vapour from evaporating pressure to condensing pressure through multi-stages to improve the COP by reducing the work of compressor, including cascade cycle systems and two-stage cycle systems. In cascade cycles, the high and low-pressure stages use different refrigerants and are coupled together through an intermediate exchanger. In a two-stage cycle, the same refrigerant is used in the entire heat pump system, so the heat exchanger between the low-pressure and high-pressure stages can be replaced with a phase separator tank (i.e., flash tank). In different designs, the flash tank can be used for intercooling^8^, sub-cooling^9^, flash gas removal^10,11^, or the combination of them^12–14^. Depending on the layout, the compression process in such systems is a two-stage compression with a certain degree of intercooling.

Citarella et al.^11^ achieved an increase of 7% in COP by using the two-stage flash gas removal configuration as compared with the case without gas bypass. Several researchers also studied the optimal condenser temperature of the two-stage cycles using ammonia, carbon dioxide^15–18^, R404A^19^, R134a^20^. Högberg and Berntsson investigated single and two-stage heat pumps using non-azeotropic mixtures and pure working fluids^21^. Lee et al.^22^ found that the coefficient of performance (COP) in modulated two-stage injection heat pump is 1.4% higher than those of the single-stage heat pump at the different weather conditions. Although two-stage heat pumps can achieve a higher COP, they are more complicated and required two compressors, and thus they are more expensive than single-stage systems. For example, they are around 30% more expensive than single-stage heat pump products in the UK market^23^.

In addition to two-stage heat pumps, a vapour injection technology has recently been developed. It can provide higher heat supply temperature at low outdoor temperatures by reducing the compressor’s discharge temperature, in a more economical way than a two-stage heat pump^24^. Essentially, most vapour injection heat pumps are built upon two-stage heat pump cycle with an internal heat exchanger as sub-cooler. Instead of two compressors, vapour injection heat pumps use a single multi-stage compressor with an added injection port between the compression chambers^25^. Use of this technology could provide more than 30% improvement at high-pressure ratios compared to conventional single-stage compressor^26^. COP improvement compared to single stage is also noticeably higher in deep frozen conditions, where it reaches between 18.4% and 23.4% at outdoor temperatures of −20 °C, to provide heat at indoor temperature between 12.5 °C and 18 °C^27^. However, these compressors require a control strategy for the best efficiency especially at low outdoor temperatures^28,29^. Typically traditional two-stage compressors with a double cylinder applications are limited due to the high cost of the compressor, the complexity of controlling oil return and the overall size of the system^27^. A conventional vapour injection heat pump cycle also features either an internal heat exchanger or a flash tank, which is placed between the condenser, the main expansion valve and the vapour injection suction line^30^. Comparison of performance and cost between the two concludes that the flash tank cycle as the most suitable option, despite the internal heat exchanger having a wider injection operating range^24,31,32^. This added feature also involves the need of control and refrigerant charge management strategy in the system^7^.

ASHPs, regardless the type of thermodynamic cycle, are all subject to evaporator frosting. Ice build-up occurs on the fin surface of the evaporator when the ambient air temperature drops below 6 °C and relative humidity is higher than 67%^33^. During frosting the heating capacity drops and the power consumption increases, reducing the COP^34,35^.

Several defrosting methods are commonly used by ASHPs in the market, including: compressor shutdown^36^, electric heating^37^, hot water spray^38^, hot gas bypass^39^, and reverse cycle defrosting (RCD)^40^. RCD is the most widely used defrosting method due to the short defrost time and minor system modifications required. After ice is detected on the outdoor unit of a RCD system, the heat pump switches to a reverse cycle mode to extract heat from the indoor unit (condenser) to defrost the outdoor unit (evaporator). The indoor unit is switched off while the compressor is turned on during defrosting. Although the compressor still consumes electricity, there is no heating supply from the heat pump, and a backup heater is needed (normally an electrical immersion heater in a hot water tank) to provide heating. After the reverse cycle defrosting, a long recovery time can be needed before an acceptable indoor temperature is reached. Despite improvements made to RCD^41,42^ by modifying components^41,43^ and implementing control measures^44,45^ to reduce the duration of defrosting, the lack of heat supply and the substantial power consumption during defrosting remain an important issue^39^.

Vapour injection heat pumps are also subject to frosting^28,46^. During the defrost cycle the vapour injection is closed in most cases, making the system switch back to a regular cycle, losing the benefits of vapour injection^47^. Notably a study on reverse defrost cycle using vapour injection concludes that the main improvement would be a shortened defrosting duration by 7.75%^48^. The defrosting of current ASHPs could consume 5–17% of the systems’ heat production annually, e.g., in the UK^43–45,49^.

Recently, there is a growing interest in integrating heat storage with heat pumps to improve its flexibility, which currently focuses on storing part of the produced heat at the supply temperature^50^ to maintain continuous heating or to provide a heat source for RCD^51–57^. Such methods can shorten defrosting times, with reduced impacts on indoor temperatures during defrosting. However, the addition of a phase change material heat storage could increase heat pump cost by 30% and increase power consumption compared to a regular RCD^53^. Similar results have been reported when using a phase change material to store heat rejected by the compressor casing^56^.

As summarised above, despite growing interest in using heat pumps for decarbonisation, significant challenges remain, including high capital costs, inefficient defrosting, interrupted heating, low COPs at high supply temperature, and the inflexibility to provide heat for both space heating and domestic hot water using a single device. Radical innovations are therefore required to improve their cost-effectiveness in order to compete with the fossil-fuel-based heating technologies.

In order to address these challenges, this paper proposes and demonstrates a flexible heat pump cycle. It integrates a heat storage, either latent or sensible, into a traditional Evans-Perkins cycle to recover, store, and reuse the sub-cooling heat carried by the hot liquid refrigerant exiting the condenser. The flexible heat pump technology achieves substantial COP improvement, and also potentially provides a new energy-saving defrosting method if it is applied to ASHPs.

## Results

### The flexible heat pump concept

**Fig. 1:**
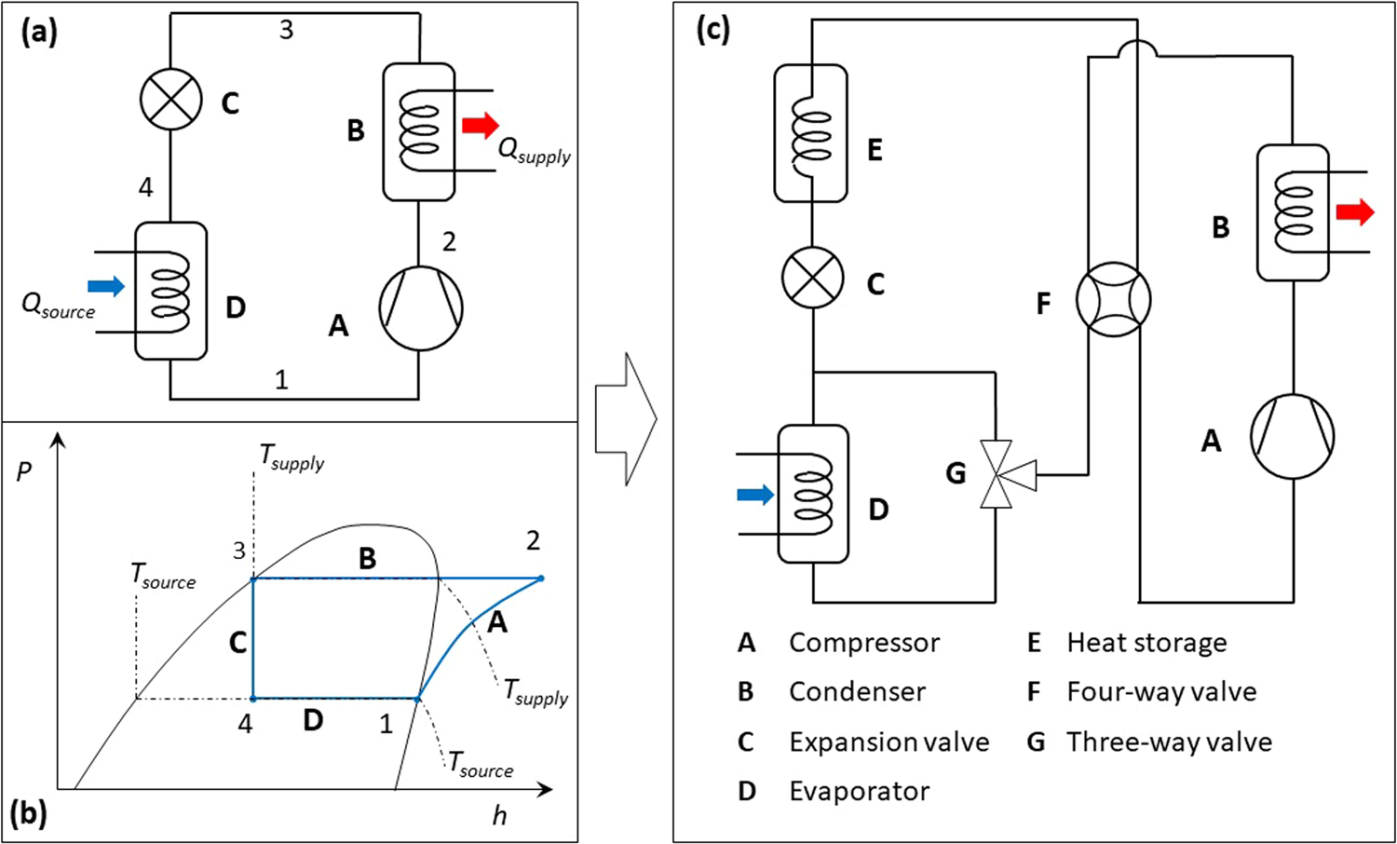
The flexible heat pump cycle in comparison with the standard single-stage heat pump cycle (i.e., Evans-Perkins cycle). **a** standard single-stage vapour compression cycle heat pump; **b** the corresponding p-h diagram (refrigerant R134a) showing that substantial unused thermal energy contained in the hot liquid refrigerant exiting the condenser is degraded during throttling process 3-4; **c** the proposed flexible heat pump cycle with heat storage to recover part of the sub-cooling heat and then use it as an ancillary heat source.

Most ASHPs in the market are single-stage systems with a heat supply temperature up to 65 °C^23^. As shown in Fig. 1a, the single-stage heat pumps are built upon the Evans-Perkins vapour-compression cycle that was originally developed for refrigeration in 1834^58^. Its basic design consists of a compressor (A), a condenser (B), an expansion valve (C), and an evaporator (D).

The corresponding pressure-enthalpy (p-h) diagram of an ideal Evans-Perkins cycle is shown in Fig. 1b. The compressor extracts low pressure and low temperature refrigerant vapour from the evaporator and compresses it to the condensing pressure. The high pressure and high temperature refrigerant vapour discharged by the compressor de-superheats and condenses in the condenser. The obtained hot liquid refrigerant leaves the condenser at the condensing temperature and still contains a substantial amount of sensible heat.

The patented flexible heat pump cycle (PCT publication reference number: WO2022069581A1) can recover, store, and utilise this heat for a number of power-saving applications. As shown in Fig. 1c, a heat storage (E), latent or sensible, is introduced to the Evans-Perkins cycle as a sub-cooler to recover and store the heat carried by the hot refrigerant liquid exiting the condenser. The rest of system is the same set of components as conventional heat pumps. The components are connected via a four-way valve (F) and a three-way valve (G) so the refrigerant can be directed to flow through the system in different orientations to charge or discharge the heat storage.

**Fig. 2:**
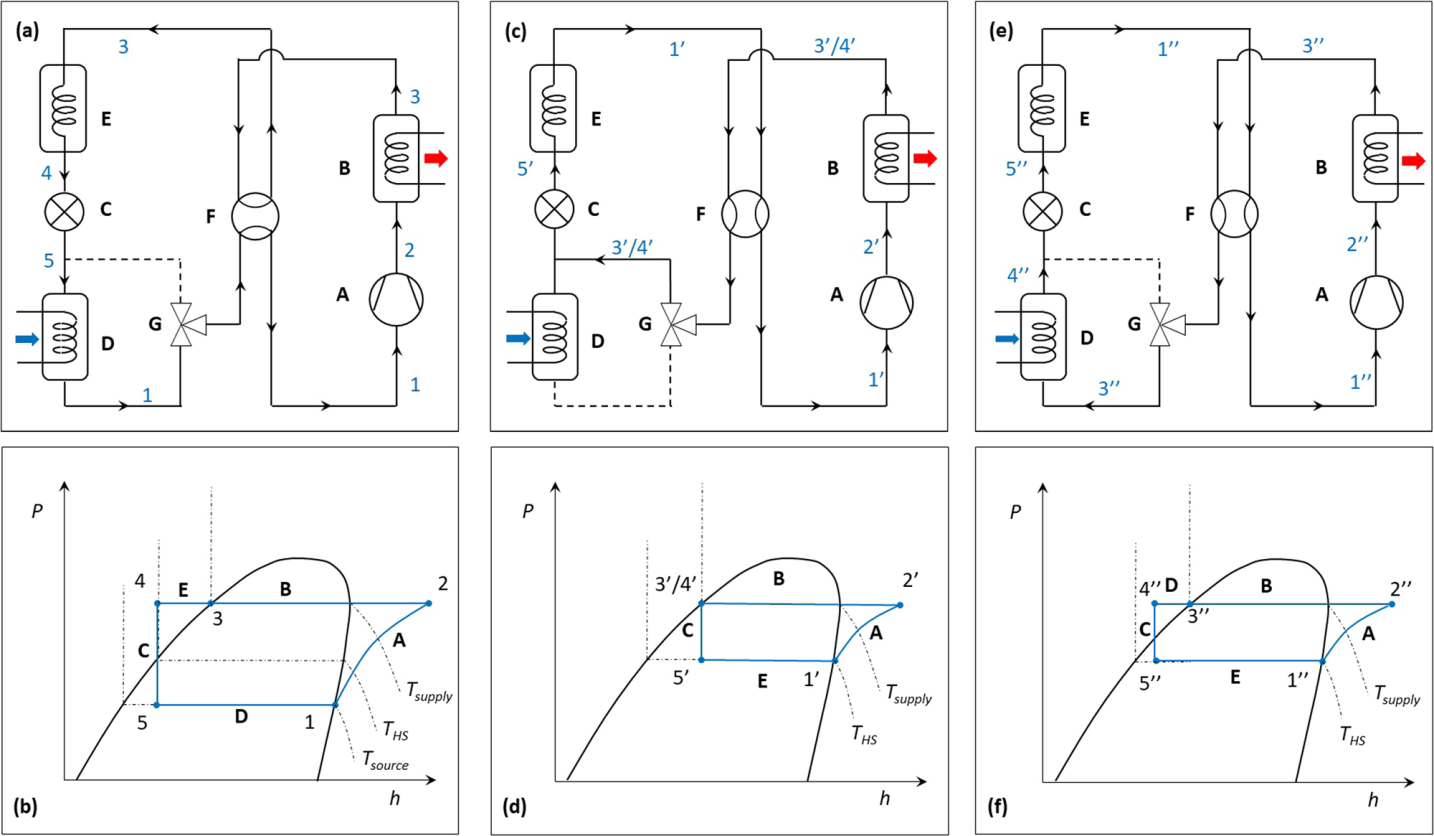
Quasi-two-stage operation of the flexible heat pump cycle and its thermodynamic diagrams. **a** Charging mode. **b** p-h diagram of the charging mode. **c** Discharging mode – can be used for either spacing heating with higher COP or domestic hot water with higher temperature. **d** p-h diagram of the discharging mode. **e** Discharging with defrosting mode. **f** p-h diagram of the discharging with defrosting mode. The connections indicated by dashed lines are deactivated.

The proposed flexible heat pump system has several beneficial operational modes, as shown in Fig. 2, with the working principles described as follows.

In-cycle heat recovery using heat storage: Setting the four-way valve (F) and three-way valve (G) as shown in Fig. 2a, the compressor (A) compresses the low-pressure vapour from the evaporator (D) to condensing pressure. The high-pressure vapour de-superheats and condenses in the condenser (B) to supply heat. The hot refrigerant liquid releases heat to the heat storage (E) and is sub-cooled. The sub-cooled liquid refrigerant is then throttled within the expansion valve (C) to the evaporating pressure. The produced mixture then fully evaporates in the evaporator (D) to absorb heat from heat source. The corresponding pressure-enthalpy (p-h) diagram is shown in Fig. 2b. Process ‘3-4’ represents the heat recovery and sub-cooling process by the heat storage.

Unlike the standard Evans-Perkins cycle as shown in Fig. 1a, the heat storage recovers some subcooling heat from the hot liquid refrigerant and stores it at a temperature higher than the evaporating temperature for the heat pump to use later.

Quasi-two-stage operation for COP improvement or heat supply temperature lift: After the heat storage is charged, the valves can be set to Fig. 2c to switch the heat pump to discharging mode, with the p-h diagram shown in Fig. 2d. The refrigerant from the compressor (A) flows through the condenser (B) to the expansion valve (C), bypassing the evaporator (D). After expansion, heat is absorbed from the heat storage (E) which now acts as an ‘evaporator’ temporarily. The produced vapour is then compressed to the condensing pressure.

The recovered thermal energy, stored at an elevated temperature in the heat storage, is used as a temporary heat source for the heat pump during discharging mode, which leads to two beneficial applications: 1) to increase COP and reduce compressor power, by reducing the source-sink temperature difference when the heat supply temperature remains the same as the charging mode; 2) to provide a higher heat supply temperature than the charging mode, by maintaining the source-sink temperature difference or COP, e.g. for domestic hot water supply.

In effect using the heat storage as an evaporator allows a single-stage heat pump to periodically act as the high stage of a two-stage system. This process has been denoted as a quasi-two-stage system hereafter. An example of this could be a heat pump which supplies underfloor heating at 35 °C during charging mode and switches to provide domestic hot water, normally over 65 °C, during discharging mode.

Defrosting and uninterrupted heating supply: For ASHP applications, the discharge mode of the flexible heat pump cycle can be used for defrosting the evaporator. The charging mode remains the same as shown in Fig. 2a. The discharging mode with defrosting is shown in Fig. 2e. During discharging mode, hot liquid refrigerant leaving the condenser is passed through the evaporator releasing heat for defrosting, and the resulting subcooled liquid is throttled as normal. The resulting p-h diagram is shown in Fig. 2f. As the ice on the evaporator gradually melts, the temperature of the refrigerant exiting the evaporator (D) gradually increases, such that point ${4}^{{\prime} {\prime} }$ moves towards point ${3}^{{\prime} {\prime} }$.

In essence, this approach utilises the same sensible heat carried by the hot refrigerant leaving the condenser for defrosting, while maintaining heat pump operation and heating supply. This could potentially eliminate the need for backup heaters.

### Simulations

As described in the Methods section, a comprehensive theoretical model has been established for the proposed flexible heat pump to study its benefits compared with the conventional systems. Based on the theoretical model, this section presents the numerical simulations used to assess the advantages of the flexible heat pump cycle with latent or sensible heat storage. The thermophysical properties are obtained using REFPROP V9.0^59^.

**Fig. 3:**
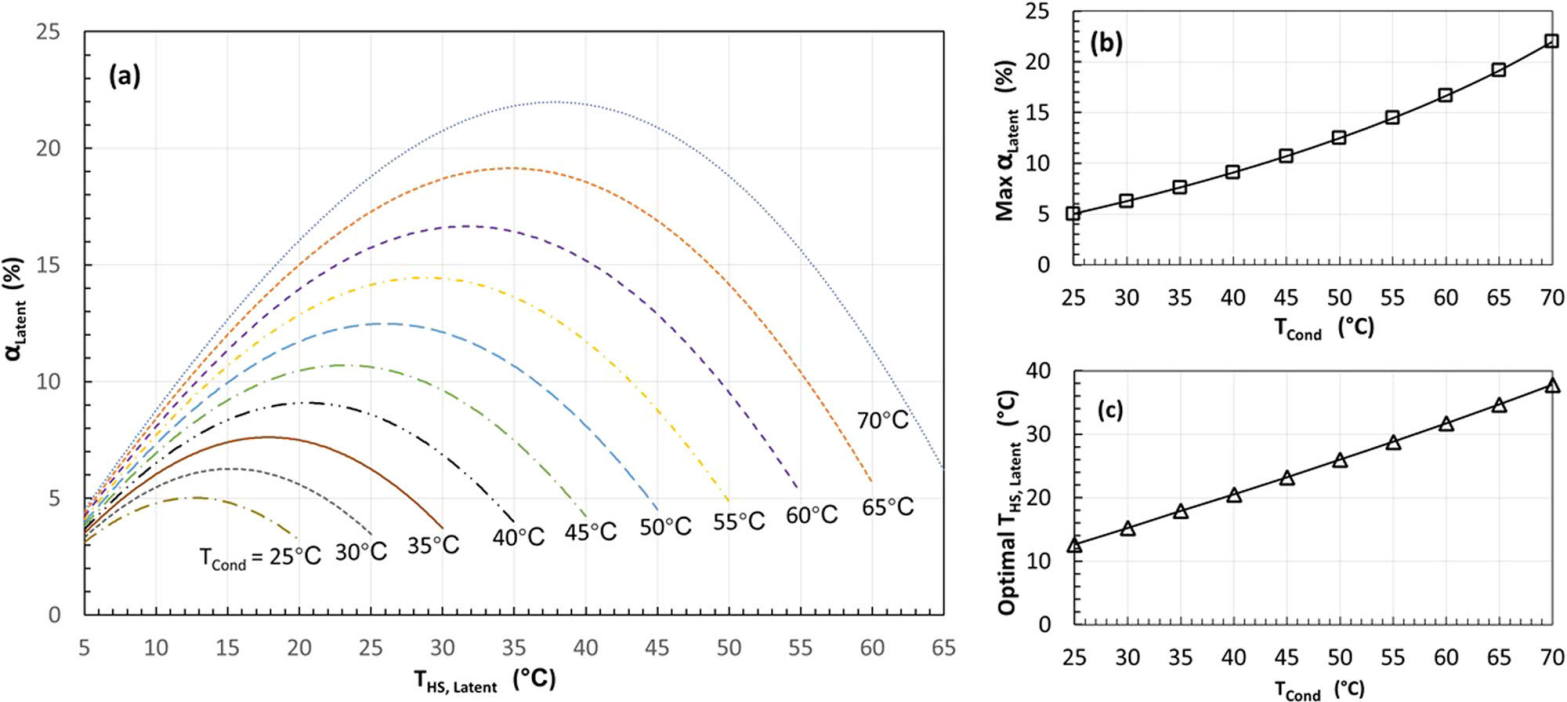
Simulation results of the flexible heat pump cycle with a latent heat storage for an evaporating temperature of 0 °C when the condensing temperature varies from 25 to 70 °C. **a** COP improvement ${{{{{{\rm{\alpha }}}}}}}_{{{{{{\rm{Latent}}}}}}}$ varies as the latent heat storage temperature varies for each given condensing temperature. **b** The maximum COP improvement increases as the condensing temperature increases. **c** The corresponding optimal temperature of the latent heat storage varies, increasing with the condensing temperature.

Latent heat storage: The simulated results using a latent heat storage are presented in Fig. 3. The selected refrigerant is R134a which is a mainstream refrigerant for heat pumps in the market. The evaporating temperature is set as 0 °C, the condensing temperatures varies from 25 to 70 °C with a step of 5 °C. The phase change temperature of the latent heat storage, ${T}_{{{{{{\rm{HS}}}}}},{{{{{\rm{Latent}}}}}}}$, varies from 5 °C above the evaporating temperature to 5 °C below the condensing temperature. As shown in Fig. 3a, for each given condensing temperature (${T}_{{{{{{\rm{cond}}}}}}}$), there is a maximum COP improvement, ${\alpha }_{{{{{{\rm{Latent}}}}}}}$, corresponding to an optimal ${T}_{{{{{{\rm{HS}}}}}},{{{{{\rm{Latent}}}}}}}$. As shown in Fig. 3b, the max ${\alpha }_{{{{{{\rm{Latent}}}}}}}$ increases from 5% to around 22% when ${T}_{{{{{{\rm{cond}}}}}}}$ increases from 25 to 70 °C. As shown in Fig. 3c, the optimal ${T}_{{{{{{\rm{HS}}}}}},{{{{{\rm{Latent}}}}}}}$ increases with ${T}_{{{{{{\rm{cond}}}}}}}$. For example, the calculated max ${\alpha }_{{{{{{\rm{Latent}}}}}}}$ is 19.14%, and the corresponding optimal ${T}_{{{{{{\rm{HS}}}}}},{{{{{\rm{Latent}}}}}}}$ is 34.7 °C when ${T}_{{{{{{\rm{cond}}}}}}}=65$ °C.

According to Fig. 2b and Eq. (19), for a given condensing temperature ${T}_{{{{{{\rm{cond}}}}}}}$, the charging rate increases as ${T}_{{{{{{\rm{HS}}}}}},{{{{{\rm{Latent}}}}}}}$ decreases, implying that more heat can be recovered, the downside is a lower storage temperature. As a result, according to Fig. 2d, the discharging mode will operate at a higher temperature lift due to a lower ${T}_{{{{{{\rm{HS}}}}}},{{{{{\rm{Latent}}}}}}}$, leading to higher compression power and thus lower COP. These two effects compete with each other, leading to an optimal ${T}_{{{{{{\rm{HS}}}}}},{{{{{\rm{Latent}}}}}}}$ and a maximum ${\alpha }_{{{{{{\rm{Latent}}}}}}}$ for each given ${T}_{{{{{{\rm{cond}}}}}}}$ as shown in Fig. 3a.

According to Fig. 2b and Eq. (19), for a given ${T}_{{{{{{\rm{HS}}}}}},{{{{{\rm{Latent}}}}}}}$, the higher the condensing temperature (i.e., ${T}_{3}$), the higher the charging rate, the more heat can be recovered. As a result, the system can operate on the discharging mode with higher COP for longer time, leading to a higher COP improvement ${\alpha }_{{{{{{\rm{Latent}}}}}}}$ according to Eq. (25).

**Fig. 4:**
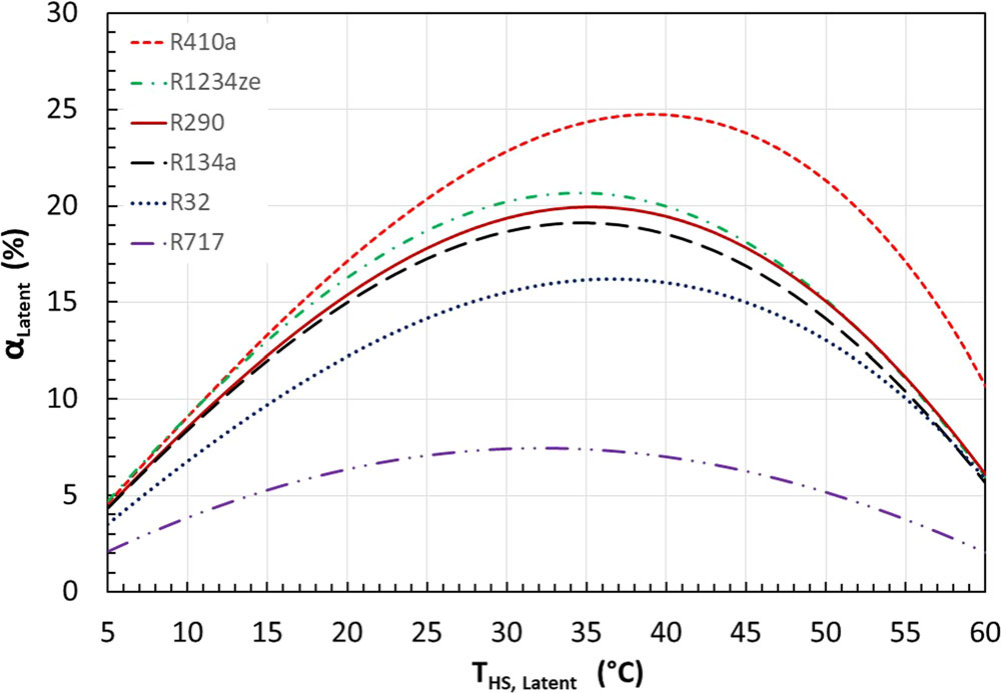
The COP improvement of the flexible heat pump cycle varies for different refrigerants. In this case study, the condenser temperature is 65 °C and the evaporating temperature is 0 °C. Six common refrigerants for heat pump applications were tested.

Effect of refrigerants: Fig. 4 shows how different commonly used refrigerants affect the flexible heat pump cycle. The condensing and evaporating temperatures are set as 65 and 0 °C, respectively. It can be seen that the highest improvement of COP can be achieved using R410a with a maximum improvement of 24.7% at ${T}_{{{{{{\rm{HS}}}}}},{{{{{\rm{Latent}}}}}}}$ of 39 °C. R1234ze (a low global warming potential replacement of R134a) could theoretically achieve a maximum COP improvement of 20.6% at ${T}_{{{{{{\rm{HS}}}}}},{{{{{\rm{Latent}}}}}}}$ of 34 °C. Similarly, the maximum COP improvement using R290 is around 20% and the corresponding optimal heat storage temperature ${T}_{{{{{{\rm{HS}}}}}},{{{{{\rm{Latent}}}}}}}$ is around 35 °C. For R32, the maximum COP improvement is around 16% at ${T}_{{{{{{\rm{HS}}}}}},{{{{{\rm{Latent}}}}}}}$ of 36 °C. However, the maximum COP improvement is only 7.47% for Ammonia. It should be noted the ranking in Fig. 4 shows the COP improvement, but not the absolute average COP using different refrigerants.

**Fig. 5:**
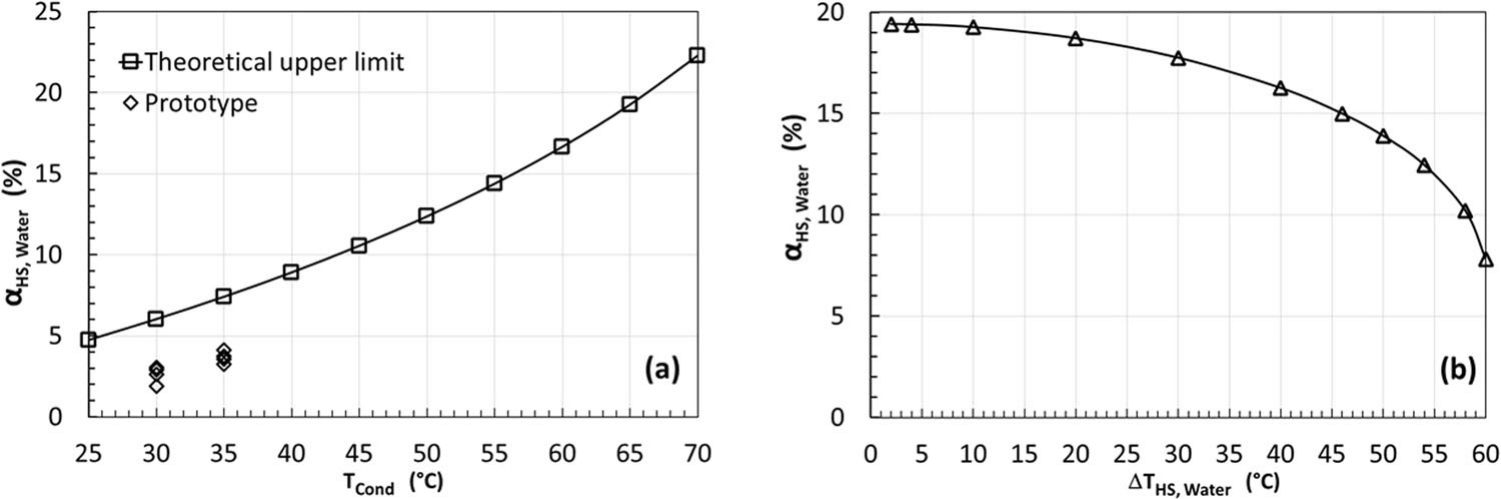
The COP improvement of the flexible heat pump cycle with a sensible heat storage using water as storage medium. The water temperature in the heat storage changes between 5 °C below and 5 °C above the optimal temperatures as found in Fig. 3c when latent heat storage is employed. **a** The COP improvement increases as the condensing temperature increases. **b** For a given condensing temperature at 65 °C, the optimal latent heat storage temperature is 34.7 °C according to Fig. 3c. The temperature of water in the heat storage tank varies from ($34.7-\triangle {{{{{\rm{T}}}}}}/2$) to ($34.7+\triangle {{{{{\rm{T}}}}}}/2$). The COP improvement increases as $\triangle {{{{{\rm{T}}}}}}$ decreases.

The COP improvement difference is mainly attributed to the portion time that the flexible heat pump can operate on the discharging mode that has a higher COP. The charging rate to the heat storage is expressed as Eq. (2), while the discharging rate can be expressed as Eq. (7). It should be noted that the mass flow rates are different for the charging and discharging modes. Considering all these factors, the operational time ratio between the discharging and charging modes is derived as Eq. (27). Table 1 summarises the calculated ${\beta }_{{{{{{\rm{Latent}}}}}}}$ of the optimal point of each curve as shown in Fig. 4. The higher this time ratio, the longer the discharging operation, and thus the higher the COP improvement. The ranking in Table 1 agrees with that in Fig. 5. As shown in the table, refrigerant R410a has the highest ${\beta }_{{{{{{\rm{Latent}}}}}}}$ of 0.46, while ammonia has the lowest ${\beta }_{{{{{{\rm{Latent}}}}}}}$ ratio of 0.15.

**Table 1:** The ratio of operational time between discharging and charging modes for the six selected refrigerants.

Refrigerant	R410a	R1234ze	R290	R134a	R32	R717
${\beta }_{{{{{{\rm{Latent}}}}}}}$	0.46	0.40	0.39	0.38	0.31	0.15

Sensible heat storage (Water): A list of cases using a water tank as the heat storage medium has been conducted and the results are presented in Fig. 5. The assumptions are the same as before except that the water temperature in the heat storage changes between 5 °C below and 5 °C above the optimal temperatures that are already found in Fig. 3c. Despite the calculations being independent of the heat storage capacity, a water tank with 25 kg is used in simulations to compare with the experimental results.

As shown in Fig. 5a, as the condensing temperature increases from 25 to 70 °C, the COP improvement ${\alpha }_{{{{{{\rm{HS}}}}}},{{{{{\rm{Water}}}}}}}$ increases from 4.7% to 22.2%, which is almost the same as the results shown in Fig. 3b when a latent heat storage is used. This can be attributed to the fact that the temperatures become less sensitive around the optimal temperature as shown in Fig. 3a.

To check the effect of water temperature range, a case study with a condensing temperature of 65 °C has been conducted. According to Fig. 3c, the optimal latent heat storage temperature is 34.7 °C. Hence, the temperature of water in the heat storage varies from ($34.7-\frac{\triangle T}{2}$) to ($34.7+\frac{\triangle T}{2}$), while $\triangle T$ varies from 2 to 60 °C in the simulations. The calculated results are presented in Fig. 5b. It can be seen that ${\alpha }_{{{{{{\rm{HS}}}}}},{{{{{\rm{Water}}}}}}}$ increases as the $\triangle T$ decreases. It increases to around 19.4 when $\triangle T$ reduces to 2 °C, which is similar to the maximum COP improvement for the same case with a latent heat storage at 34.7 °C as shown in Fig. 3b. This finding has implications for practical systems, with more cost-effective water based sensible heat systems having comparable performance to latent heat storage.

### Experiments

The experimental prototype was initially constructed as a proof-of-concept system and is being used to systematically demonstrate the various operational modes. The performance of the system operating under a quasi-two-stage mode for power reduction has been presented here.

**Fig. 6:**
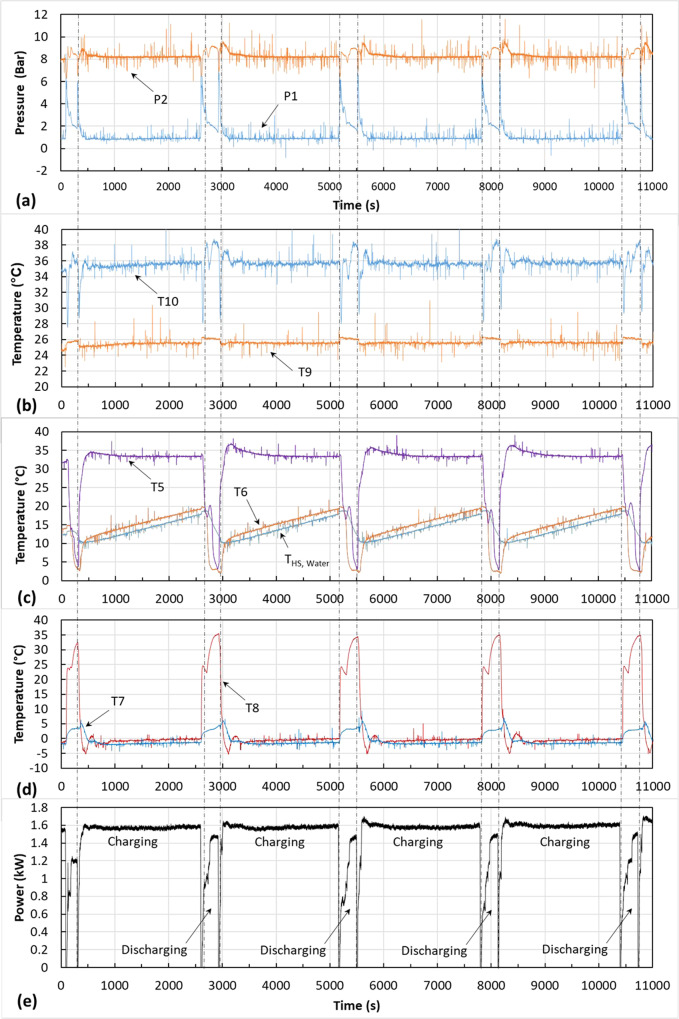
Measured temperatures, pressures, and power during 4 cycles of charging/discharging operation of the flexible heat pump prototype. **a** Pressures at the inlet (P1) and outlet (P2) of the compressor. **b** Water temperature at the inlet (T9) and outlet (T10) of the condenser. **c** Refrigerant temperature at the inlet and outlet of the heat storage and the water temperature in the heat storage water tank. **d** Refrigerant temperature at the inlet and outlet of the evaporator. **e** Compressor power, showing lower power consumption during discharging while the heating capacity of the heat pump remains constant at 2.5 kW.

The evaporating temperature target set point was ~0 °C and two condensing temperatures have been tested, 30 and 35 °C. The heating capacity is maintained at around 2.5 kW during both charging and discharging. The water temperature in the heat storage tank varies from 10 to 20 °C during charging and discharging. A set of sample results (${T}_{{{{{{\rm{cond}}}}}}}=35$ °C) are shown in Fig. 6.

Figure 6a shows the pressures at the inlet (P1) and outlet (P2) of the compressor. After initial start up the system was run for four charging/discharging cycles. Figure 6b shows the heat supply temperature (T10) is maintained at around 35 °C, with a minor increase during the discharging mode.

Figure 6c shows the refrigerant temperatures at the inlet and outlet of the heat storage and the water temperature in the heat storage tank. During charging, the hot refrigerant from the condenser (T5) is at around 35 °C. The water temperature (${T}_{{{{{{\rm{HS}}}}}},{{{{{\rm{Water}}}}}}}$) in the tank increases steadily from about 10 to 20 °C. The refrigerant leaves the heat storage at T6. The system switches to discharging mode when ${T}_{{{{{{\rm{HS}}}}}},{{{{{\rm{Water}}}}}}}$ reaches about 20 °C. During discharging, the hot liquid refrigerant from the condenser passes through the evaporator to release heat for defrosting and is sub-cooled. After the throttling process through expansion valve V6, it enters the heat storage at T6 which is now lower than the water temperature (${T}_{{{{{{\rm{HS}}}}}},{{{{{\rm{Water}}}}}}}$). It absorbs heat and leaves the heat storage at T5. Please note that T5 remains above the water temperature for a short period during switching because of thermal inertia.

Figure 6d shows the refrigerant temperatures at the inlet and outlet of the evaporator. During charging, the refrigerant from the expansion valve V5 enters the evaporator at T7 slightly below 0 °C, absorbs heat from air and leaves the evaporator at T8. During discharging with defrosting, the hot liquid refrigerant from the condenser enters the evaporator at T8 and releases heat to warm the evaporator up, and then leaves the evaporator at T7 which gradually increases from around 0 to 5 °C. Ice has been observed during the charging mode and subsequently defrosted during the discharging mode. The results qualitatively demonstrated that the hot liquid refrigerant exiting the condenser can be used for evaporator defrosting. However, the current prototype has limited humidity control of the environmental closure, which does not allow for an extensive range of environmental conditions to be tested. Further experimental research will focus on fully testing and demonstrating the proposed defrosting method.

Figure 6e shows the measured compressor power during the charging/discharging cycles. The average compressor power during four charging modes is around 1.56 kW. The calculated COP is 1.6 with a fixed heating capacity of around 2.5 kW. During discharging, the water temperature in the heat storage (Fig. 6b) decreases rapidly as it discharges heat. Consequently, the compressor power increases during discharging. The calculated average compressor power is 1.1 kW during discharging. Maintaining 2.5 kW heating capacity, the COP is 2.24. According to Eq. (25), the average COP during four charging/discharging cycles is calculated as 1.66. Using Eq. (26), the improvement of COP is calculated as 3.7%.

The COP improvement of four charging/discharging cycles shown in Fig. 6 is plotted on Fig. 5a. The same process as above was carried out at a condensing temperature of 30 °C and is also plotted in Fig. 5a. It should be noted that the experimental tests involve some evaporator defrosting, but the simulations shown in Fig. 5a bypassed the evaporator. Defrosting consumes some thermal energy and reduces the discharging time, and thus reduces the COP improvement. Nevertheless, although the measured COP improvement is only around 50% the theoretical upper limit, the trend agrees with the simulated ones.

## Discussion

As predicted by the ideal model, the flexible heat pump can operate on a quasi-two-stage mode to achieve an average COP up to 20% higher than conventional single-stage heat pumps under same operational conditions, depending on the heat supply temperature and working fluids. It should be noted that this is the theoretical upper limit and real systems will achieve less improvement.

A prototype built with off-the-shelf parts, with a small water tank as heat storage, has demonstrated 3.7% COP improvement when the heat production temperature is only 35 °C, achieving around 50% of the calculated theoretical upper limit under the same condensing temperature as shown in Fig. 5a. The COP improvement increases as the heat supply temperature increases almost linearly. As indicated by projection shown in Fig. 5 (i.e., around 50% of the theoretical upper limit), up to 10% COP improvement may be achieved as the heat production temperature increases to 65 °C in real systems.

The COP improvement is strongly affected by the portion of time that the flexible heat pump can operate on the discharging mode that has a higher COP. The higher the time ratio ${\beta }_{{{{{{\rm{Latent}}}}}}}$, the longer the discharging operation, and thus the higher the COP improvement. Therefore, refrigerants with a high specific heat in liquid state and a small latent heat of evaporation could achieve better COP improvement using the flexible heat pump cycle.

Both latent and sensible heat storage can deliver similar COP improvement, but a sensible heat storage using water is considered to be the most cost-effective solution. The cost of a small water tank heat storage is marginal, but the COP improvement is substantial.

For ASHP applications, the discharging mode can directly use the hot liquid refrigerant exiting the condenser for defrosting whilst maintaining continuous heating supply, which could further save energy compared with current ASHPs. The experimental results qualitatively demonstrated the hot liquid can provide heat to the evaporator, showing its potential for defrosting. Further numerical and experimental research is required to fully demonstrate and exploit this potential.

Limitation of the theoretical modelling: As described in Section 3, idealised thermodynamic models are used to find the theoretical maximum COP improvement using the flexible heat pump cycle. Such a theoretical maximum gives us a basis to gauge the performance of the prototype against what the possible maximum might be. Furthermore, the proposed flexible heat pump cycle can be applied to all heat pumps regardless of what the heat source is. In order to keep the model generic, only the conditions of the refrigerant side of the evaporator are considered. As a result, this approach has neglected the change of heat transfer conditions within the evaporator during the charging and discharging modes.

Comparing with the standard Evan-Perkins heat pump cycle, the amount of heat recovered by the heat storage during the charging mode of the flexible heat pump cycle needs to be compensated by an increase of heat transfer within the evaporator. As a result, the predicted maximum COP improvement by the above simulations may be slightly reduced. A more advanced model that considers all potential losses should be developed to provide more accurate calculations, which is however beyond the scope of this paper.

## Conclusion

This paper reports an invention of the flexible heat pump which integrates heat storage into the conventional vapour-compression cycle to recover the sensible heat that is contained in the hot liquid refrigerant leaving the condenser. The recovered heat is then used as an ancillary source for the heat pump operation periodically, improving the system’s overall COP. Furthermore, the flexible heat pump cycle, through the in-cycle heat storage, potentially enables wider applications for utilising intermittent, temperature-changing, low-grade heat sources, such as external waste heat recovery and further integration with systems such as solar thermal energy collector.

## Methods

### Theoretical model

Idealised theoretical models of the flexible heat pump concept have been established and are used to determine the theoretical upper limits of the flexible heat pump cycle. Establishing these limits provides a basis to compare with the developed experimental prototype. Either sensible or latent heat storage can be used. Latent heat storage has a theoretical fixed temperature for storage while sensible heat storage has a varying temperature profile. We start with a more general approach using sensible heat storage, and then simplify the model to simulate latent heat storage.

The assumptions include:

1. Heat transfer takes place under isothermal condition in all heat exchangers and the heat storage.
2. Compressor has 100% isentropic efficiency.
3. No heat and pressure losses through pipes and heat exchangers.
4. No change in condenser heating capacity.
5. Assume 100% round trip efficiency for the heat storage.

According to Fig. 2b, the heating capacity of the condenser can be expressed as

1 $${\dot{Q}}_{{{{{{\rm{c}}}}}}}={\dot{m}}_{{{{{{\rm{r}}}}}}}\left({h}_{2}-{h}_{3}\right).$$

For sensible heat storage, as temperatures ${T}_{3}$ and ${T}_{1}$ remain constant, specific enthalpies ${h}_{1},{h}_{2}$ and ${h}_{3}$ are all independent of time. Therefore, heating capacity ${\dot{Q}}_{{{{{{\rm{c}}}}}}}$, compressor power ${\dot{W}}_{{{{{{\rm{comp}}}}}}}$, and ${{COP}}_{{{{{{\rm{charge}}}}}}}$ are independent of time.

However, ${T}_{4}$ varies between the evaporating temperature ${T}_{5}$ and the condensing temperature ${T}_{3}$ during charging. Therefore, the heat charging rate ${\dot{Q}}_{{{{{{\rm{charge}}}}}}}$ to the heat storage can be expressed as

2 $${\dot{Q}}_{{{{{{\rm{charge}}}}}}}={\dot{m}}_{{{{{{\rm{r}}}}}}}({h}_{3}-{h}_{4}),$$

where the mass flow rate of refrigerant ${\dot{m}}_{{{{{{\rm{r}}}}}}}$ remains constant, but ${h}_{4}$ is a function of ${T}_{4}$ which is a function of time. The total heat stored by the heat storage during the charging time ${\triangle t}_{{{{{{\rm{charge}}}}}}}$ can be calculated as

3 $${Q}_{{{{{{\rm{charge}}}}}}}={\int }_{0}^{{\triangle t}_{{{{{{\rm{charge}}}}}}}}{\dot{m}}_{{{{{{\rm{r}}}}}}}({h}_{3}-{h}_{4}){dt}.$$

The power consumed by the compressor ${\dot{W}}_{{{{{{\rm{comp}}}}}}}$ can be calculated as

4 $${\dot{W}}_{{{{{{\rm{comp}}}}}}}={\dot{m}}_{{{{{{\rm{r}}}}}}}({h}_{2}-{h}_{1}).$$

As a result, the heat pump’s COP can be expressed as

5 $${{COP}}_{{{{{{\rm{charge}}}}}}}=\frac{{\dot{Q}}_{{{{{{\rm{c}}}}}}}}{{\dot{W}}_{{{{{{\rm{comp}}}}}}}}=\frac{{h}_{2}-{h}_{3}}{{h}_{2}-{h}_{1}}.$$

It should be highlighted that subcooling does not affect the inlet and outlet conditions of the compressor as long as the evaporating and condensing conditions are given. As a result, the subcooling does not affect either the heat production in the condenser as shown in Eq. (1) or the compressor power as shown in Eq. (4). Therefore, as shown in Eq. (5), the heat recovery by the heat storage (i.e., sub-cooling) does not affect the system’s COP during the charging mode. For this reason, the charging mode of the flexible heat pump cycle has the same COP as a conventional single heat pump cycle operating under the same temperature conditions.

During discharging, the heat storage acts as an ‘evaporator’, providing a temporary heat source. As the heat storage discharges heat, its temperature drops. According to Fig. 2d, ${T}_{1{\prime} }={T}_{5{\prime} }$, which both drop accordingly. Consequently, the pressure ratio of the compressor increases resulting in an increase in compressor’s discharge temperature ${T}_{2{\prime} }$. Therefore, ${h}_{1{\prime} }$, ${h}_{2{\prime} }$ and ${h}_{5{\prime} }$ are all functions of time.

The heating capacity of the condenser during discharging mode can be expressed as

6 $${\dot{Q}}_{{{{{{\rm{c}}}}}}{\prime} }={\dot{m}}_{{{{{{\rm{r}}}}}}{\prime} }({h}_{2{\prime} }-{h}_{3{\prime} }).$$

According to the assumption, ${\dot{Q}}_{{{{{{\rm{c}}}}}}{\prime} }={\dot{Q}}_{{{{{{\rm{c}}}}}}}$ as defined in Eq. (1).

The discharging rate of heat storage can then be expressed as

7 $${\dot{Q}}_{{{{{{\rm{discharge}}}}}}}={\dot{m}}_{{{{{{\rm{r}}}}}}{\prime} }({h}_{1{\prime} }-{h}_{5{\prime} }).$$

Here, ${\dot{m}}_{r{\prime} }$ is the time-dependent mass flow rate of refrigerant during discharging mode.

Compressor work during discharging mode can be written as

8 $${\dot{W}}_{{{{{{\rm{comp}}}}}}{\prime} }={\dot{m}}_{{{{{{\rm{r}}}}}}{\prime} }({h}_{2{\prime} }-{h}_{1{\prime} }).$$

Here, ${\dot{m}}_{{{{{{\rm{r}}}}}}{\prime} }$, ${h}_{2{\prime} }$ ${h}_{1{\prime} }$, and ${h}_{5{\prime} }$ are all functions of time, as a result, ${\dot{Q}}_{{{{{{\rm{discharge}}}}}}}$, ${\dot{W}}_{{{{{{\rm{comp}}}}}}{\prime} }$ are also functions of time. Hence, total heat discharged from the heat storage during discharging ${\triangle t}_{{{{{{\rm{discharge}}}}}}}$ can be calculated as

9 $${Q}_{{discharge}}={\int }_{0}^{{\triangle t}_{{{{{{\rm{discharge}}}}}}}}{\dot{Q}}_{{{{{{\rm{discharge}}}}}}}{dt}={\int }_{0}^{{\triangle t}_{{{{{{\rm{discharge}}}}}}}}{\dot{m}}_{{{{{{\rm{r}}}}}}{\prime} }({h}_{1{\prime} }-{h}_{5{\prime} }){dt}.$$

The total compressor work during the discharge can be calculated as

10 $${W}_{{comp}{\prime} }={\int }_{0}^{{\triangle t}_{{{{{{\rm{discharge}}}}}}}}{\dot{W}}_{{{{{{\rm{comp}}}}}}{\prime} }{dt}={\int }_{0}^{{\triangle t}_{{{{{{\rm{discharge}}}}}}}}{\dot{m}}_{{{{{{\rm{r}}}}}}{\prime} }({h}_{2{\prime} }-{h}_{1{\prime} }){dt}.$$

Total heat discharged must be the same as the total heat charged to the heat storage, therefore

11 $${\int }_{0}^{{\triangle t}_{{{{{{\rm{discharge}}}}}}}}{\dot{m}}_{{{{{{\rm{r}}}}}}{\prime} }({h}_{1{\prime} }-{h}_{5{\prime} }){dt}={\int }_{0}^{{\triangle t}_{{{{{{\rm{charge}}}}}}}}{\dot{m}}_{{{{{{\rm{r}}}}}}}({h}_{3}-{h}_{4}){dt}.$$

As ${\dot{Q}}_{{{{{{\rm{c}}}}}}{\prime} }={\dot{Q}}_{{{{{{\rm{c}}}}}}}$, the total heat production during discharge mode is

12 $${Q}_{{{{{{\rm{c}}}}}}{\prime} }={\dot{Q}}_{{{{{{\rm{c}}}}}}}{\triangle t}_{{{{{{\rm{discharge}}}}}}}.$$

As a result, the system’s COP during the discharging mode is

13 $${{COP}}_{{{{{{\rm{discharge}}}}}}}=\frac{{Q}_{{{{{{\rm{c}}}}}}{\prime} }}{{W}_{{{{{{\rm{comp}}}}}}{\prime} }}=\frac{{\dot{Q}}_{c}{\triangle t}_{{{{{{\rm{discharge}}}}}}}}{{\int }_{0}^{{\triangle t}_{{{{{{\rm{discharge}}}}}}}}{\dot{m}}_{{{{{{\rm{r}}}}}}{\prime} }({h}_{2{\prime} }-{h}_{1{\prime} }){dt}}.$$

According to Eq. (12), the total heat production during one charging/discharging cycle is calculated as

14 $${Q}_{{{{{{\rm{total}}}}}}}={\dot{Q}}_{{{{{{\rm{c}}}}}}}\triangle {t}_{{{{{{\rm{charge}}}}}}}+{\dot{Q}}_{c}\triangle {t}_{{{{{{\rm{discharge}}}}}}}={\dot{Q}}_{c}\left(\triangle {t}_{{{{{{\rm{charge}}}}}}}+\triangle {t}_{{{{{{\rm{discharge}}}}}}}\right).$$

Total compressor work ${W}_{{{{{{\rm{total}}}}}}}$ during one charging/discharging cycle can be calculated as

15 $${W}_{{{{{{\rm{total}}}}}}}={\dot{W}}_{{{{{{\rm{comp}}}}}}}\triangle {t}_{{{{{{\rm{charge}}}}}}}+{\int }_{0}^{{\triangle t}_{{{{{{\rm{discharge}}}}}}}}{\dot{m}}_{{{{{{\rm{r}}}}}}{\prime} }({h}_{2{\prime} }-{h}_{1{\prime} }){dt}.$$

The average COP during one charge/discharge cycle can be calculated as

16 $$\overline{{COP}}=\frac{{Q}_{{{{{{\rm{total}}}}}}}}{{W}_{{{{{{\rm{total}}}}}}}}=\frac{{\dot{Q}}_{{{{{{\rm{c}}}}}}}\left(\triangle {t}_{{{{{{\rm{charge}}}}}}}+\triangle {t}_{{{{{{\rm{discharge}}}}}}}\right)}{{\dot{W}}_{{{{{{\rm{comp}}}}}}}\triangle {t}_{{{{{{\rm{charge}}}}}}}+{\int }_{0}^{{\triangle t}_{{{{{{\rm{discharge}}}}}}}}{\dot{m}}_{{{{{{{\rm{r}}}}}}}^{{\prime} }}\left({h}_{{2}^{{\prime} }}-{h}_{{1}^{{\prime} }}\right){dt}}.$$

According to Eq. (4) and the discussion above, subcooling does not affect the COP of a heat pump. Hence, a conventional heat pump operating at the same evaporating/condensing temperatures would have the same COP as shown in Eq. (5). Therefore, we can use the COP of the charging mode as the benchmark. As a result, the COP improvement $\alpha$ of the proposed flexible heat pump system can be calculated as

17 $$\alpha =\frac{\overline{{COP}}-{{COP}}_{{{{{{\rm{charge}}}}}}}}{{{COP}}_{{{{{{\rm{charge}}}}}}}}\times 100 \% .$$

The ratio of operational time between the discharging and charging modes $\beta$ can be expressed as

18 $$\beta =\frac{\triangle {t}_{{{{{{\rm{discharge}}}}}}}}{\triangle {t}_{{{{{{\rm{charge}}}}}}}},$$

and it can be calculated once Eq. (11) is solved.

When a latent heat storage is used, the charging rate to the heat storage is calculated using Eq. (2). The total heat charged to the heat storage can be calculated using Eq. (3) as

19 $${Q}_{{{{{{\rm{charge}}}}}}}={\dot{m}}_{{{{{{\rm{r}}}}}}}({h}_{3}-{h}_{4}){\triangle t}_{{{{{{\rm{charge}}}}}}},$$

where ${\dot{m}}_{{{{{{\rm{r}}}}}}}$, ${h}_{3},$ and ${h}_{4}$ are independent of time.

Power input to the compressor can be calculated using Eq. (4). As a result, the heat pump’s COP on charging mode can be calculated using Eq. (5).

During discharging, the heating capacity of the condenser can be written as Eq. (6). According to the assumption, ${\dot{Q}}_{{{{{{\rm{c}}}}}}{\prime} }={\dot{Q}}_{{{{{{\rm{c}}}}}}}$, and they can be calculated using Eq. (6) and Eq. (1), respectively.

The heat storage will function as an ‘evaporator’ during discharging mode, the discharging rate can be expressed as Eq. (7). Since ${\dot{m}}_{{{{{{\rm{r}}}}}}{\prime} }$, ${h}_{1{\prime} }$, and ${h}_{5{\prime} }$ remain unchanged during discharging mode, the total heat discharged by the heat storage can be calculated using Eq. (9) as

20 $${Q}_{{{{{{\rm{discharge}}}}}}}={\dot{m}}_{{{{{{\rm{r}}}}}}{\prime} }({h}_{1{\prime} }-{h}_{5{\prime} }){\triangle t}_{{{{{{\rm{discharge}}}}}}}.$$

Similarly, the compressor power can be written as Eq. (8), and the total compressor work during discharging can be calculated using Eq. (10) as

21 $${W}_{{{{{{\rm{comp}}}}}}{\prime} }={\dot{m}}_{{{{{{\rm{r}}}}}}{\prime} }({h}_{2{\prime} }-{h}_{1{\prime} }){\triangle t}_{{{{{{\rm{discharge}}}}}}}.$$

Therefore, the COP of the system during the discharge can be expressed as

22 $${{COP}}_{{{{{{\rm{discharge}}}}}},{{{{{\rm{latent}}}}}}}=\frac{{Q}_{{{{{{\rm{c}}}}}}{\prime} }}{{W}_{{{{{{\rm{comp}}}}}}{\prime} }}=\frac{{\dot{Q}}_{{{{{{\rm{c}}}}}}{\prime} }}{{\dot{W}}_{{{{{{\rm{comp}}}}}}{\prime} }}=\frac{{h}_{2{\prime} }-{h}_{3{\prime} }}{{h}_{2{\prime} }-{h}_{1{\prime} }}.$$

To maintain repeatable balance, the heat discharged must equal the heat charged as the heat storage is assumed to have 100% round trip efficiency, hence

23 $${\dot{Q}}_{{{{{{\rm{discharge}}}}}}}\triangle {t}_{{{{{{\rm{discharge}}}}}}}={\dot{Q}}_{{{{{{\rm{charge}}}}}}}\triangle {t}_{{{{{{\rm{charge}}}}}}}.$$

Total heat production of the heat pump during one cycle of charging/discharging can be calculated as Eq. (14).

Total compressor work can be calculated according to Eq. (15) as

24 $${W}_{{{{{{\rm{total}}}}}}}={\dot{W}}_{{{{{{\rm{comp}}}}}}}\triangle {t}_{{{{{{\rm{charge}}}}}}}+{\dot{W}}_{{{{{{\rm{comp}}}}}}{\prime} }\triangle {t}_{{{{{{\rm{discharge}}}}}}}.$$

Therefore, the average COP of the heat pump during one charging/discharging cycle can be calculated as

25 $${\overline{{COP}}}_{{{{{{\rm{Latent}}}}}}}=\frac{{Q}_{{{{{{\rm{total}}}}}}}}{{W}_{{{{{{\rm{total}}}}}}}}=\frac{{\dot{Q}}_{c}\left(\triangle {t}_{{{{{{\rm{charge}}}}}}}+\triangle {t}_{{{{{{\rm{discharge}}}}}}}\right)}{{\dot{W}}_{{{{{{\rm{comp}}}}}}}\triangle {t}_{{{{{{\rm{charge}}}}}}}+{\dot{W}}_{{{{{{\rm{comp}}}}}}{\prime} }\triangle {t}_{{{{{{\rm{discharge}}}}}}}}.$$

Therefore, the COP improvement of the flexible heat pump system can be calculated as

26 $${\alpha }_{{{{{{\rm{Latent}}}}}}}=\frac{{\overline{{COP}}}_{{{{{{\rm{Latent}}}}}}}-{{COP}}_{{{{{{\rm{charge}}}}}}}}{{{COP}}_{{{{{{\rm{charge}}}}}}}}\times 100 \% .$$

Substituting Eqs. (19) and (20) into Eq. (18), the ratio of operational time between discharging and charging modes when latent heat storage is employed can calculated as

27 $${\beta }_{{{{{{\rm{Latent}}}}}}}=\frac{\triangle {t}_{{{{{{\rm{discharge}}}}}}}}{\triangle {t}_{{{{{{\rm{charge}}}}}}}}=\frac{{\dot{m}}_{{{{{{\rm{r}}}}}}}({h}_{3}-{h}_{4})}{{\dot{m}}_{{{{{{\rm{r}}}}}}{\prime} }({h}_{1{\prime} }-{h}_{5{\prime} })}$$

### Experimental design

**Fig. 7:**
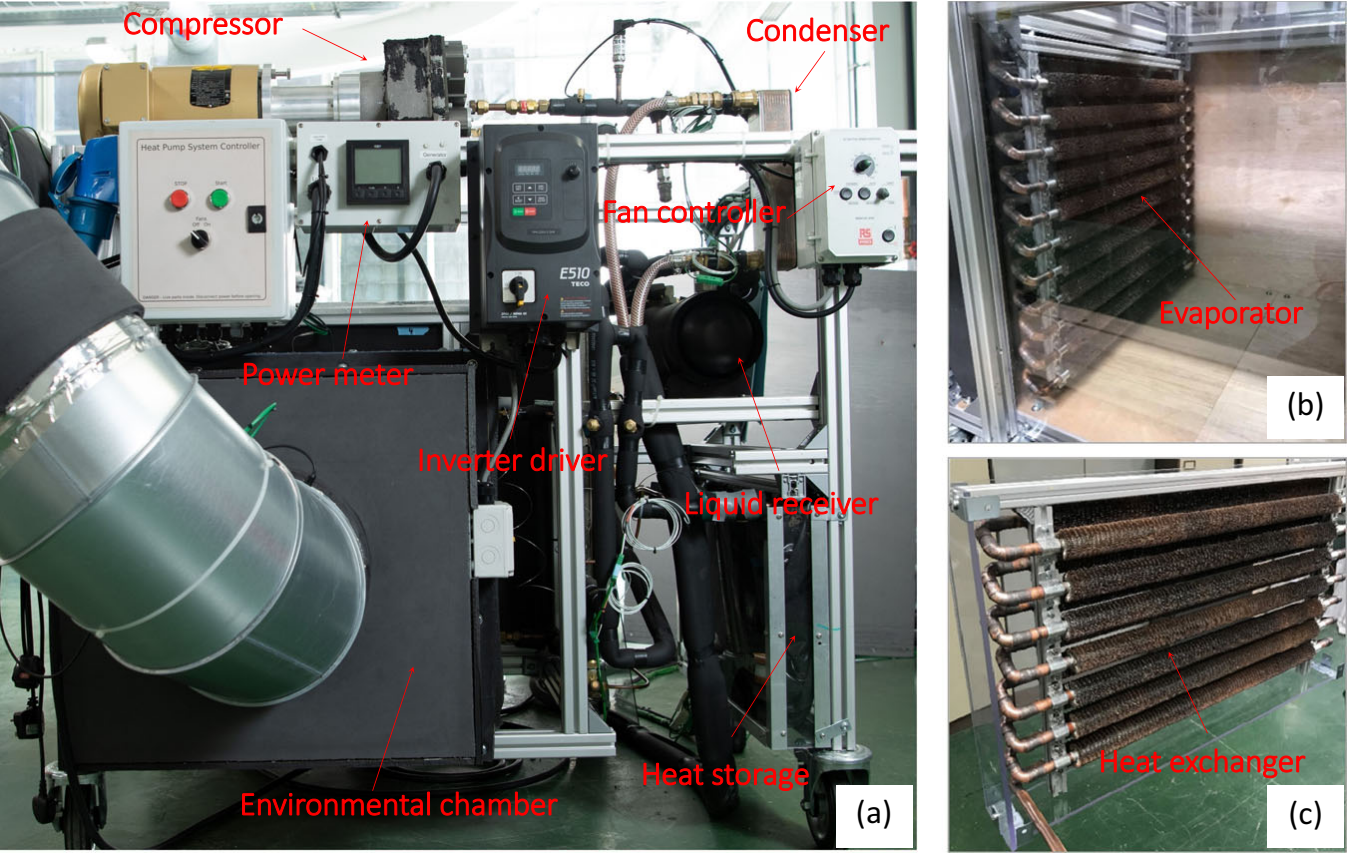
Photographs of the flexible heat pump prototype. **a** A photo of the flexible heat pump prototype. **b** An environmental chamber that houses the evaporator, where air temperature and humidity level can be adjusted and controlled. **c** The heat exchanger made of finned copper tubes for heat storage water tank, where refrigerant flows inside the tubes and water outside the copper tubes.

As shown in Fig. 7, the prototype of the proposed flexible heat pump comprises a 1-kW oil-free scroll compressor (Air squared Ltd, Model: P15H022A-A01). It is controlled via an inverter drive, with the compressor and inverter power measured with a power metre, and thus the measured power includes both the power losses of the inverter and the power consumption by the compressor. The condenser, a brazed plate heat exchanger, transfers heat from the refrigerant to a water loop. The return water temperature is controlled using a chiller. Excesses refrigerant is retained within the liquid receiver (LR).

**Fig. 8:**
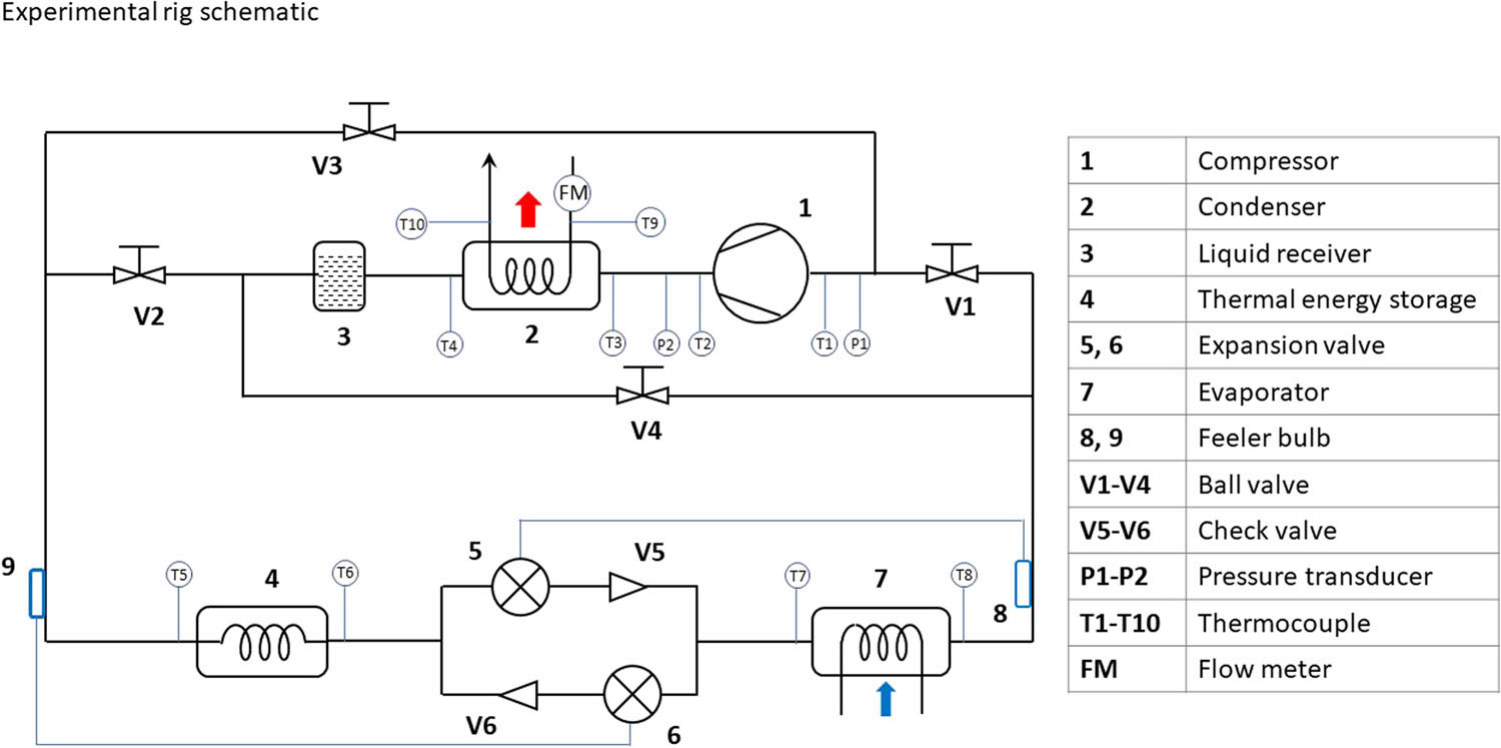
The schematic of the flexible heat pump prototype and instrumentation. Charging mode: Open V1 and V2, close V3 and V4, feeler bulb 8 controls expansion valve V5. Discharging and defrosting mode: Close V1 and V2, open V3 and V4, feeler bulb 9 controls expansion valve V6.

Figure 8 presents the schematic and instrumentation of the prototype. The four-way control valve shown in Fig. 2 has been replaced with four manually operated ball valves (V1–V4), for control flexibility reasons. The heat storage system comprises a 27-litre tank with water as the storage medium. The refrigerant passes through a series of wire wound copper tubes immersed within the tank. It is estimated that 25 littler of water was contained in the tank after adding the heat exchanger. The expansion process is controlled via two thermostatic expansion valves with two one-way check valves (V5, V6). The evaporator is comprised of a series of wire wound copper tubes located within an environmental enclosure. The environmental enclosure circulates air using a fan. The temperature of the air is controlled using a bank of finned tubes which recirculate temperature-controlled water.

Two operational modes can be achieved by setting the control valves V1–V4 with different combinations as described below:

1. Charging mode: Open V1 and V2, close V3 and V4, feeler bulb 8 controls expansion valve V5.
2. Discharging and defrosting mode: Close V1 and V2, open V3 and V4, feeler bulb 9 controls expansion valve V6.

Temperatures of the refrigerant flow have been measured at the inlet and outlet of the compressor, condenser, heat storage, expansion valve and evaporator using Type K thermocouples T1–T8 as shown in Fig. 8. The pressures at the inlet and outlet of the compressor are measured using two pressure transducers (Omega PX319). The water temperature at the inlet and outlet of the condenser are measured using two Type K thermocouples T9-T10, and its mass flow rate is measured using a flow metre (Titan – FT2 turbine flow metre: 065).

It should be noted that, the objective of this prototype is to prove the flexible heat pump concept, rather than validating the ideal theoretical model. It was built with off-the-shelf parts which are not necessarily optimised. Nevertheless, as both charging and discharging modes work on the same set of components, it is sufficient for us to demonstrate the benefits of energy recovery, the higher COP of the discharging mode, and the working principle of the proposed waste-heat driven defrosting method. Due to the limitation of the compressor, the system has been only tested with a condensing temperature up to 35 °C.

The experimental procedures are described as follows:

1. Before heat pump start up: the environmental enclosure fan is turned on and the temperature in the enclosure is reduced from ambient to target temperature. The water heating loop supplying the condenser is brought up to temperature and the desired flow rate set. The small pump which circulates water around the thermal storage water tank is turned on and left on for the duration of the testing.
2. Heat pump start up: The heat pump is set to discharging mode initially in order to reduce the heat storage water tank temperature from ambient to target low cycle switch temperature. This is achieved by switching off the environmental enclosure fan and closing valve V1 and V2, ensuring V3 and V4 are open. The compressor is switched on and speed is controlled manually to maintain desired heat input to the condenser heated water loop. Once target water tank temperature is reached the compressor is switched off.
3. Discharging to Charging mode switch: With the compressor off, valves V1 and V2 are opened which results in pressure equalisation of the system. Valve V3 and V4 are then closed. The environmental enclosure fan is switched on and the compressor is engaged. Compressor speed is increased gradually until the desired heating water temperature is reached.
4. Charging mode: Once the desired supply temperature is met the system is monitored and left to run, charging the thermal storage water tank. Once the target high switch temperature is reached, the compressor and the environmental fan are switched off.
5. Charging to discharging mode switch: Valves V3 and V4 are opened equalising the pressure in the system. Valves V1 and V2 are closed. The compressor is switched on with the speed manually controlled for desired output, discharging the heat storage. Once the low target temperature is reached the compressor is stopped and the cycle is switched as above. The process is repeated for duration of testing.

## Data Availability

The datasets generated and/or analysed during the current study are available within the paper. Other materials and data are available from the corresponding author, Z.Y., upon reasonable request.
